# High Doses of Vitamin D and Specific Metabolic Parameters in Type 2 Diabetes Patients: Systematic Review

**DOI:** 10.3390/nu16223903

**Published:** 2024-11-15

**Authors:** Filip Max, Andrea Gažová, Juraj Smaha, Martin Jankovský, Tomáš Tesař, Peter Jackuliak, Martin Kužma, Juraj Payer, Ján Kyselovič

**Affiliations:** 1 Department of Organisation and Management of Pharmacy, Faculty of Pharmacy, Comenius University, Odbojarov 10, 832 32 Bratislava, Slovakia; 2 Institute of Pharmacology and Clinical Pharmacology, Faculty of Medicine, Comenius University Bratislava, 813 72 Bratislava, Slovakia; andrea.gazova@fmed.uniba.sk; 3 5th Department of Internal Medicine, Faculty of Medicine, University Hospital, Comenius University, Ruzinovska 6, 826 06 Bratislava, Slovakia; smaha1@uniba.sk (J.S.); mrtn.jnkvsk@gmail.com (M.J.); peter.jackuliak@fmed.uniba.sk (P.J.); martin.kuzma@fmed.uniba.sk (M.K.); payer@ru.unb.sk (J.P.); jan.kyselovic@fmed.uniba.sk (J.K.)

**Keywords:** diabetes mellitus type 2, T2DM, cholecalciferol, vitamin D, systematic review, metabolic parameters, fasting blood glucose, blood pressure, serum calcium, parathormone

## Abstract

**Background/Objectives:** Type II diabetes mellitus (T2DM) is recognized as a condition of mild chronic inflammation, marked by increased levels of acute-phase proteins and various inflammatory indicators. These inflammatory substances, along with inflammation of adipose tissue and the secretion of adipocytokines, can contribute to insulin resistance and β cell dysfunction. By influencing both innate and adaptive immunity, vitamin D can inhibit the production of inflammatory cytokines and help mitigate the low-grade chronic inflammation associated with T2DM. Several strategies have been proposed to increase vitamin D levels effectively and safely, but the recent and strong ones have common tactics. Short-term high doses increase the level acutely, and long-term lower doses maintain sufficient levels. **Methods:** The aim of our work was to determine and verify the effectiveness of high doses of vitamin D to safely increase its level in patients with type 2 diabetes mellitus, as well as the effect of these doses on selected metabolic parameters. Data from 20 studies (vitamin D group *n* = 612, and control group *n* = 592) regarding the influence of vitamin D supplementation with doses above 4000 IU on serum 25(OH)D, fasting blood glucose (FBG), hemoglobin A1c (HbA1c), blood pressure, serum calcium, and parathormone were pooled. **Results:** Vitamin D supplementation significantly improved serum 25(OH)D levels, with an average increase after intervention versus baseline at 177.09%. Our studies suggest that vitamin D supplementation may benefit various parameters in T2DM patients, including glycemic control, blood pressure, and PTH levels. **Conclusions:** Vitamin D supplementation may have beneficial effects on various parameters in type 2 diabetes patients, including glycemic control, blood pressure, and parathormone levels. However, the results are only sometimes consistent across all studies. Further examination is needed.

## 1. Introduction

Since the elucidation of vitamin D’s chemical structure in 1930 by Nobel Prize winner Adolf Otto Reinhold Windaus, building on insights from earlier researchers, significant advancements have been made in vitamin D studies [1]. Initially, the focus was on its metabolic effects on bones, highlighting the essential function of vitamin D and its metabolites in maintaining calcium balance and bone metabolism. However, with the identification of 25(OH)D in 1968, followed by 1,25-hydroxyvitamin D [1,25(OH)2D], research broadened to encompass various fields, including immune disorders, infections, cancer, and cardiovascular conditions [2]. Vitamin D plays a crucial role in the regulatory processes of the immune system; it also enhances the intestinal absorption of phosphate and inhibits its elimination by the kidneys. While vitamin D’s importance for bone health is widely recognized, it is merely one facet of the molecule’s diverse functions. Pleiotropy is the expression of several traits by one gene, and thus, a gene or a pair of genes with multiple phenotypic manifestations has a pleiotropic effect [3].

T2DM is regarded as a condition of mild chronic inflammation, marked by increased levels of acute-phase proteins and various inflammatory markers. These inflammatory substances, along with inflammation of adipose tissue and the secretion of adipocytokines, can contribute to insulin resistance and dysfunction of β cells [4]. Through its influence on both innate and adaptive immunity, vitamin D can inhibit the production of inflammatory cytokines and diminish the mild chronic inflammation associated with T2DM [5]. The regulatory mechanisms of vitamin D in connection with innate immunity have been known for a long time [6]. The increase in the antimicrobial activity of macrophages and monocytes is a consequence of the higher production of cathelicidin antimicrobial peptide (CAMP) and defensin β2, which is due to 1,25(OH)2D3. The increase in CAMP by vitamin D is also important for the effect on other cells of the innate immune system [7]. In addition to macrophages and monocytes, it also increases autophagy, chemotaxis, and phagolysosomal fusion in cells of innate immunity. Vitamin D can modulate the innate immune system. 1,25(OH)2D3 strengthens the function of the physical barrier of intestinal epithelial cells and also increases the ability of immune cells to phagocytose [8]. The role of vitamin D is also significant in the largest organ of the immune system, the intestine. It helps maintain intestinal homeostasis and integrity, affects the composition of the intestinal microbiome, and reduces intestinal permeability. This helps reduce inflammation [9]. The effect of vitamin D on adaptive immunity is mainly related to T and B lymphocytes. Vitamin D supports the differentiation and activation of T-lymphocytes, especially T-helper cells (Th). Th cells play a key role in regulating the immune response. Vitamin D helps the transition of T-lymphocytes to T-regulatory cells (Treg), which contribute to the suppression of excessive immune reactions and ensure immune tolerance [10]. Vitamin D can inhibit the synthesis of pro-inflammatory Th1, Th9, or Th22 cytokines and also stimulates the synthesis of anti-inflammatory Th2 cytokines. Vitamin D also stimulates B-lymphocytes to produce antibodies. It also reduces the levels of pro-inflammatory cytokines (such as interleukin 6 (IL-6) and tumor necrosis factor-alpha (TNF-α)) and promotes the production of anti-inflammatory cytokines (such as interleukin 10 (IL-10)). It is reported that 1,25(OH)2D3 helps inhibit pro-inflammatory cytokines such as interleukin 2 (IL-2), interferon gamma (IFN γ), and TNF α in Th1, interleukin 9 (IL-9) in Th9 and interleukin 17 (IL-17), and interleukin 21 (IL-21) in Th17. Conversely, 1,25(OH)2D3 helps to stimulate anti-inflammatory factors, such as interleukin 4 (IL-4), interleukin 5 (IL-5), IL-9 or interleukin 13 (IL-13) in Th2 cells, or IL-10 in Treg cells [11]. In B cells, vitamin D can inhibit some immunoglobulins, such as immunoglobulin M (IgM) and immunoglobulin G (IgG). Also, in this way, vitamin D contributes to a balanced immune response, which is important for the prevention of chronic inflammatory diseases such as T2DM [12]. Furthermore, the presence of vitamin D receptor (VDR) in skeletal muscle cells suggests a potential role for vitamin D in the master regulatory system of glucose homeostasis in muscle tissue. Vitamin D stimulates the expression of the human insulin receptor gene and, therefore, modulates the action of insulin and increases insulin sensitivity [5]. To better understand the pathophysiological consequences of vitamin D deficiency and its relationship to diabetes, it is necessary to recall the molecular mechanisms by which vitamin D acts. Genomic effects are mediated by VDR, which belongs to the superfamily of steroid and retinoid nuclear receptors [13]. Up to three percent of the genes making up the human genome are regulated by vitamin D, more precisely by its active form 1,25(OH)2D3. VDR is expressed in many tissues and cells, most of which are vitamin D targets, including pancreatic β-cells [14].

The still-prevailing pandemic of vitamin D deficiency has not diminished significantly in recent years. High-quality RCTs and meta-analyses currently indicate that ~40% of Europeans are vitamin D-deficient (<50 nmol/L), and 13% are severely deficient (<30 nmol/L) [15]. Seventeen percent of adolescents and 32% of young adults from Australia were vitamin D-deficient [16]. The prevalence of low vitamin D status in Africa was 17.31%, with a cut-off of serum 25(OH)D concentration less than 30 nmol/L, 34.18% for a cut-off of <50 nmol/L, and 58.54% of <75 nmol/L [17]. In Asia, 20.93% of the population had 25(OH)D levels <25 nmol/L, 22.82% had levels <30 nmol/L, 57.69% had levels <50 nmol/L, and 76.85% had levels <75 nmol/L [18].

Despite these interesting facts, recent Endocrine Society guidelines advise against routine 25-hydroxyvitamin D 25(OH)D testing in healthy individuals and call for limiting vitamin D supplementation beyond the daily recommended intake to healthy individuals. It advises that people who may benefit from vitamin D supplementation include children aged 1–18 years, to prevent rickets and to potentially lower the risk of respiratory tract infections; pregnant women, to lower the risk for maternal and fetal or neonatal complications; adults older than 75 years, to lower the risk for mortality; and adults with prediabetes, to lower the risk for T2DM [19]. The question remains: how can blood vitamin D levels be safely and efficiently increased in a short period? The dose of vitamin D required to increase people to a minimum serum 25(OH)D of 20 ng/mL (50 nmol/L) is approximately 800 IU daily, whereas increasing people to a minimum level of 30 ng/mL (75 nmol/L) would require approximately 4000 IU daily [20]. How about optimal levels above 30 ng/mL (75 nmol/L)? Is it necessary to increase intake even more? Several strategies have been proposed to increase vitamin D levels effectively and safely, but the recent and strong ones have common tactics. Short-term high doses increase the level, and long-term lower doses acutely serve to maintain sufficient levels [21]. Central and Eastern European experts recommend a vitamin D supplementation dose of 800 to 2000 IU of vitamin D per day for adults who want to ensure a sufficient vitamin D status. These doses are also recommended for the treatment of vitamin D deficiency. Still, higher vitamin D doses (e.g., 6000 IU per day) may be used for the first 4 to 12 weeks of treatment if a rapid correction of vitamin D deficiency is clinically indicated before continuing, with a maintenance dose of 800 to 2000 IU daily [22].

Our systematic review aimed to investigate the efficacy and safety of oral cholecalciferol administration in higher doses and its effect on specific metabolic parameters in patients with T2DM.

## 2. Methods

### 2.1. Eliglibility Criteria

We followed the Preferred Reporting Items for Systematic Reviews and Meta-Analyses (PRISMA) in this systematic review [23]. We have defined the criteria for considering studies for this systematic review as follows: Population (T2DM adults); Intervention (orally administered vitamin D); Control (placebo or active comparator or no supplementation); Outcome (positive outcomes for selected clinical parameters: vitamin D (VD), glycated hemoglobin (HbA1C), blood pressure (BP; systolic (SBP), diastolic (DBP), parathormone (PTH), serum calcium (Ca^2+^), fasting blood glucose (FBG), body mass index (BMI)); Study design (randomized controlled trials (RCTs) published since 1 January 2000—double-blind, placebo-controlled (DBPC), open-label randomized controlled (RC) studies, double-blind, active comparator-controlled (DBACC), RC open-label investigation, randomized placebo-controlled trial (RPCT), prospective interventional randomized trial (PIRT)). Language—trials published in English.

### 2.2. Inclusion Criteria

We set the inclusion criteria as follows: 1. original articles of randomized controlled trials—DBPC, open-label RC studies, DBACC, RC open-label investigation, RPCT, PIRT; 2. adult patients with diagnosed T2DM; 3. patients receiving orally administered vitamin D above 4000 IU a day (inclusive) on a daily or weekly basis; and 4. trials evaluating vitamin D levels and at least two metabolic parameters, such as HbA1c, blood pressure, PTH, Ca, and FBG.

### 2.3. Exclusion Criteria

We set the exclusion criteria as follows: trials assessing vitamin D below 4000 IU a day, using a monthly dosage of vitamin D or using other than oral administration of vitamin D, experimental trials, animal model trials, prediabetes patients, patients with type 1 diabetes, gestational diabetes patients, patients under 18 years, and trials with insufficient or incomparable variables.

### 2.4. Limitations

Limitations include the observation that many studies did not account for the impact of sun exposure; some also omitted factors such as BMI or physical activity, which could have affected vitamin D levels. Additionally, the use of antidiabetic medications, insulin, or other therapies may obscure the benefits of vitamin D, particularly in studies that permitted adjustments to medication during the intervention phase. Lastly, certain trials had small sample sizes and relatively brief intervention periods.

### 2.5. Search Strategy and Data Extraction

The systematic literature search was performed on 8 February 2024 in the SCOPUS, Web of Science, and PubMed databases.

In SCOPUS we used the following search terms: TITLE-ABS-KEY (cholecalciferol OR vitamin D OR D3) AND TITLE-ABS-KEY (randomised OR randomized) AND TITLE-ABS-KEY (diabetes) AND PUBYEAR>1999 AND PUBYEAR<2025 AND (LIMIT-TO (DOCTYPE, “or”)) AND (LIMIT-TO (LANGUAGE, “English”)).

In Web of Science, search terms were ((TS = (cholecalciferol OR vitamin OR D3)) AND TS = (diabetes)) AND TS = (randomised OR randomized), Language: English, Publication date: since 2000.

However, we used the following in PubMed: (vitamind OR cholecalciferol OR D3 AND diabetes [tiab] AND ((randomizedcontrolledtrial[Filter]) AND (english[Filter]) AND (2000:2024[pdat]))) AND ((randomizedcontrolledtrial[Filter]) AND (english[Filter])).

We limited all the searches with language restrictions to English and article type to clinical trials. We identified 809 references (SCOPUS 330, PubMed 208, Web of Science 271). We screened all abstracts and removed duplicates and references, experimental trials, animal model trials, and references researching prediabetes patients, patients with type 1 diabetes, or gestational diabetes patients, and obtained 87 references. After another screening, we removed duplicates, references assessing vitamin D below 4000 IU a day or using monthly dosage of vitamin D or using other than oral administration of vitamin D, references with patients below 18 years, trials with insufficient or uncomparable variables, i.e., trials evaluating less than two of metabolic parameters of HbA1c, blood pressure, PTH, Ca^2+^, and FBG, and obtained a result of 21 references, which we studied precisely. The data collected from the studies included the year of publication, population characteristics, type of trial, vitamin D dose, intervention duration, control characteristics, number of patients, and parameters. These are in Table 1.

## 3. Results

### 3.1. Study Selection

**Figure 1:**
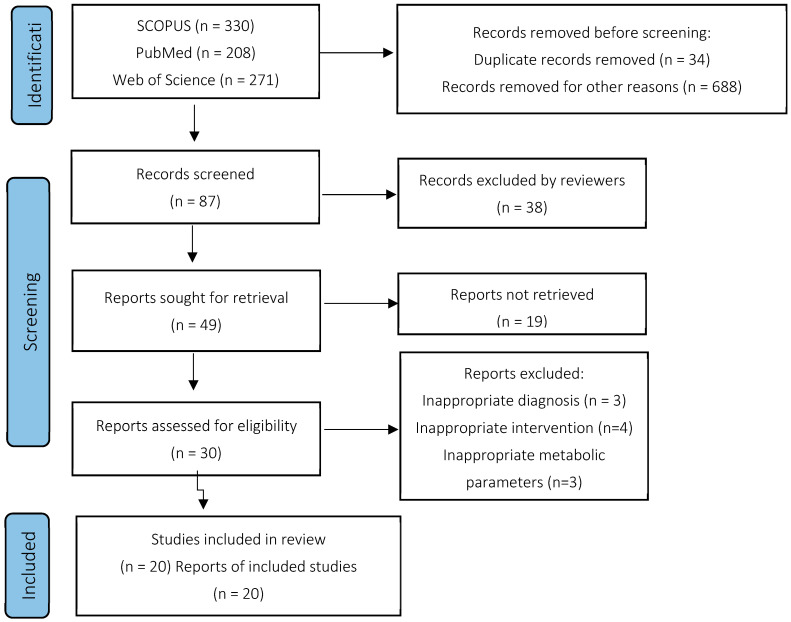
Flowchart of the search strategy and data extraction based on PRISMA 2020.

We comprehensively and systematically read 87 articles. Of these, we excluded 66 for the following reasons: incomparable data (*n*  =  38), references not meeting supplementation criteria (*n*  =  17), and trials evaluating less than two metabolic parameters, HbA1c, blood pressure, PTH, Ca^2+^, and FBG (*n*  =  11). We summarized the data extraction process in a flowchart (Figure 1).

### 3.2. Effect on Parameters

#### 3.2.1. Effect on Vitamin D Levels

The shortest duration of intervention was eight weeks, and the longest was 12 months. Out of 20 studies, 10 used supplementation of 50,000 IU of vitamin D weekly. All the studies showed that vitamin D levels increased significantly. The difference between levels after intervention and levels at baseline has been reported in %, with 17 of the studies having an increase in vitamin D levels above 100%. Not a single study showed any sign of reaching toxic levels. According to many authors, recommendations and guidelines consider optimal vitamin D levels between 40 and 60 ng/mL (100–150 nmol/L). Nine studies had average levels of vitamin D after intervention between this scale, optimal respectively (Table 2).

#### 3.2.2. Effect on HbA1c Levels

Of 18 studies, 14 showed some decrease in HbA1c levels after the intervention. Although three studies showed an increase in HbA1c after the vitamin D intervention, the highest difference from baseline was only 3.2%, which should not indicate a deterioration in blood sugar control. However, it is important to note that the standard deviation is high, possibly indicating large variability between studies (Table 2).

#### 3.2.3. Effect on Blood Pressure

Systolic and diastolic blood pressures were measured in 14 studies. In nine of them, SBP decreased after the intervention. However, the difference in baseline levels was significant in four studies. Barale et al. (2020) [26] report that one-year vitamin D supplementation, able to restore D status, significantly improves FG, HbA1c, SBP, and HDL-cholesterol levels in patients with poor-controlled T2DM and vitamin D deficiency. Still, there is a significant amount of heterogeneity between studies. The same can be said regarding results in DBP. Of those 14 studies, 9 reported a decrease in DBP, but only 3 had a decrease above 5%, which can be considered significant. Only 7 out of all 14 studies have shown a decrease in both SBP and DBP levels. It is also another example of heterogeneity in studies (Table 2).

#### 3.2.4. Effect on Parathormone

Only seven studies reported data on the measurement of PTH levels. However, all of them showed decreased PTH levels after intervention versus baseline, with an average decrease of 13.93%. However, baseline levels of PTH before supplementation in all included studies were optimal and in the reference range (1.6–6.9 pmol/L). The heterogeneity is not as significant as in previous results (Table 2).

#### 3.2.5. Effect on Serum Calcium

Out of 11 studies that measured serum calcium levels, 8 showed an increase in serum calcium levels after the intervention, and 2 of those studies showed a significant increase. One study by Al Sofiani et al. [25] showed a significant increase in serum calcium levels after the intervention, even though baseline levels were above normal range levels. On the other hand, Esfandiari et al. [33] showed that baseline levels of serum calcium were below normal range levels, and vitamin D supplementation increased those levels by 3.25% (Table 2).

#### 3.2.6. Effect on Fasting Blood Glucose

Although 15 studies reported data on FBG, only 8 showed a decrease after the intervention, while 8 showed an increase in levels. The average rate of decrease after supplementation of high doses of vitamin D was 6.08%, while the increase was 3.75%. That means that heterogeneity was significant between studies and results (Table 2).

#### 3.2.7. Effect on Body Mass Index

BMI was measured in 18 of all studies, but only 14 have been checking the effect of the intervention on it. The average baseline BMI of all included participants in vitamin D groups was 29.73, which is considered overweight. Because all patients have T2DM, a higher BMI was expected. BMI was lowered in 11 of 14 studies, and the average decrease was by 1.64%. The increase in BMI was measured in three studies with an average value of 0.46% (Table 2).

### 3.3. Risk of Bias

The included randomized studies varied in design, structure, and methodology. One study used allocation concealment, while 19 did not report on it. All studies were randomized, employing various randomization methods. Fourteen studies were double-blinded, reducing bias risk; four did not explicitly mention blinding, and two were open-label, potentially increasing bias risk.

In 15 studies, some subjects dropped out for various reasons, often unrelated to the treatment. Regarding selective reporting, 12 studies seemed to report all relevant outcomes. Two had registered protocols, and in six, assessing selective reporting bias was difficult. No study mentioned other potential biases, though four had third-party funding, with authors declaring no conflicts of interest (Table 3).

## 4. Discussion

This systematic review found that effective and short-term supplementation with doses of vitamin D above 4000 IU a day yielded some positive effects on various parameters in T2DM patients. Supplementation was safe, tolerable, and effective in increasing vitamin D levels to reach optimal scale. It was important to note that patients were older and had higher BMI, as is usual in T2DM patients, which means lower bioavailability of vitamin D. Although the findings for some metabolic parameters were not particularly strong when examining each study individually, there was considerable heterogeneity, partly due to variations in duration, participant numbers, dosages, and other factors across the studies. In contrast, we found no advantage of vitamin D supplementation in enhancing serum calcium and fasting blood glucose (FBG). Nevertheless, despite the overall results, the specific characteristics of the interventions and the populations involved affected the outcomes, leading to significant positive effects in several subgroups. Although most studies implemented methods to reduce the risk of bias, such as randomization and blinding, some studies have potential risks of bias, such as open design and participant dropout. It is important to consider these factors when interpreting the results of these studies.

Ahmadi et al. (2013) [24] and Elkassaby et al. (2014) [32] investigated the effect of vitamin D3 supplementation on proteinuria in patients with T2DM and found no significant reduction. Al-Sofiani et al. (2015) [25] examined the effect of vitamin D supplementation on glucose metabolism and inflammatory response in T2DM patients. They discovered that vitamin D improved β-cell activity but did not significantly change HbA1c or insulin sensitivity. Barale et al. (2020) [26] conducted a pilot study on poorly controlled T2DM patients and found that vitamin D supplementation improved glycemic control, lipid profile, and systolic blood pressure. Behshad et al. (2022) [27] also studied the effect of vitamin D3 on glycemic control and lipid profile in T2DM patients and found a slight decrease in HbA1c and fasting blood sugar levels. Byrn et al. (2019) [28] focused on the effect of vitamin D supplementation on cognitive function in T2DM patients but found no significant differences between high-dose and low-dose groups. Cojic et al. (2020) [29] investigated vitamin D’s anti-inflammatory and antioxidative effects in T2DM patients on metformin therapy and found that vitamin D may improve endothelial dysfunction. Ebrahimkhani et al. (2020) [30] studied the effects of combined vitamin D and curcuminoid supplementation on anthropometric measurements and blood pressure in T2DM patients. The results showed that vitamin D reduced systolic and diastolic blood pressure, while curcuminoids reduced mostly diastolic blood pressure. El Hajj et al. (2020) [31] evaluated the effect of vitamin D treatment on inflammatory markers in non-obese Lebanese patients with T2DM and found a significant reduction in hs-CRP and TNF-α concentrations. Esfandiari et al. (2019) [33] examined the effects of vitamin D3 on metabolic and inflammatory parameters in diabetic nephropathy patients and found that vitamin D supplementation reduced proteinuria and inflammatory markers. Imanparast et al. (2019) [34] examined the combined effects of chromium picolinate and vitamin D3 on insulin resistance and tumor necrosis factor-alpha in T2DM patients. They ascertained that the combination therapy helped control insulin resistance. Jorde et al. (2009) [35] found that vitamin D supplementation did not improve glycemic control in diabetic subjects with normal vitamin D levels. Kampmann et al. (2014) [36] studied the effects of high-dose vitamin D3 on insulin sensitivity, beta-cell function, and metabolic markers in T2DM patients with vitamin D insufficiency. It was found that vitamin D supplementation did not improve insulin resistance or glycemic control but might increase insulin secretion. Khan et al. (2023) [37] investigated the effects of vitamin D as an add-on therapy to metformin and teneligliptin in T2DM patients. Their results showed that vitamin D improved glycemic parameters in patients with vitamin D deficiency. Momeni et al. (2016) [38] evaluated the effect of vitamin D3 therapy on proteinuria in T2DM patients with vitamin D deficiency or insufficiency. It was observed that vitamin D supplementation decreased proteinuria in these patients. Muley et al. (2019) [39] studied the impact of vitamin D3 supplementation on anthropometric measurements and glycemic and lipid parameters in T2DM subjects. They found that vitamin D supplementation improved anthropometric and lipid parameters. Sadyia et al. (2014) [40] conducted a randomized controlled trial to study the effect of vitamin D3 supplementation on metabolic control in obese T2DM patients. It turned out that vitamin D3 normalized vitamin D status but did not affect metabolic control. Safarpour et al. (2020) [41] assessed the effects of vitamin D supplementation on SIRT1, irisin, and insulin resistance in overweight/obese T2DM patients. Their results showed that vitamin D increased SIRT1 and irisin levels and decreased HbA1c. Tabesh et al. (2015) [42] investigated the effects of calcium and vitamin D3 supplementation on anthropometric measurements and blood pressure in vitamin D-insufficient T2DM patients. It was ascertained that combined supplementation reduced BMI, hip circumference, and systolic blood pressure. Yiu et al. (2013) [43] conducted a randomized controlled trial to investigate the effect of vitamin D supplementation on endothelial function in T2DM patients. They discovered that vitamin D did not improve vascular function or biomarkers of inflammation and oxidative stress.

## 5. Conclusions

This collection of articles explores the relationship between vitamin D and type 2 diabetes mellitus. Overall, these studies suggest that vitamin D supplementation may have beneficial effects on various parameters in type 2 diabetes patients, including glycemic control, blood pressure, and parathormone levels, although the results are not always consistent across all studies. The 20 studies included in this review evaluating the role of vitamin D in type 2 diabetic patients have large variations in their study designs. Oral vitamin D supplementation has shown better effects in enhancing optimal serum 25(OH)D levels in an effective and safe way. However, it did not appear to influence fasting blood glucose or serum calcium. Additional randomized double-blinded controlled trials are needed to further refine and clarify these findings and strengthen the current evidence on the effects of vitamin D on metabolic parameters in patients with type 2 diabetes.

## Figures and Tables

**Table 1:** Specification of studies, supplementation, and patients. DBPC—double-blind, placebo-controlled, RC—randomized controlled, DBACC—double-blind, active comparator-controlled, RPCT—randomized placebo-controlled trial, PIRT—prospective interventional randomized trial, RCT—randomized controlled trial, UAE—United Arab Emirates.

Name of the First Author and Year of Publication	Population Characteristics (All Adults)	Type of Trial	Dose of Vitamin D (IU)	Duration of Intervention	Control Characteristics	Vitamin D Group (Number of Patients)	Control Group (Number of Patients)
Ahmadi 2013 [24]	Iran	DBPC	50,000/weekly	12 weeks	placebo	28	23
Al-Sofiani 2015 [25]	Saudi Arabia	DBPC	5000/daily	12 weeks	placebo	10	10
Barale 2020 [26]	Italy	open-label RC pilot study	5000/daily	12 months	no supplementation	16	14
Behshad 2022 [27]	Iran	DBPC	50,000/weekly	8 weeks	placebo	30	32
Byrn 2019 [28]	USA	DBACC	50,000/weekly	3 months	comparator (5000 IU)	15	15
Cojic 2020 [29]	Montenegro	RC open-label investigation	50,000/weekly/3 months; 14,000/weekly/3 months	6 months	no supplementation	70	70
Ebrahimkhani 2020 [30]	Iran	DBPC	50,000/weekly	3 months	placebo	17	19
El Hajj 2020 [31]	Lebanon	DBPC	30,000/weekly	6 months	placebo	45	43
Elkassaby 2014 [32]	Australia	DBPC	6000/daily	6 months	placebo	26	24
Esfandiari 2019 [33]	Iran	DBPC	50,000/weekly	8 weeks	placebo	25	25
Imanparast 2019 [34]	Iran	RPCT	50,000/weekly	4 months	placebo	23	23
Jorde 2009 [35]	Norway	RPCT	40,000/weekly	6 months	placebo	16	16
Kampmann 2014 [36]	Denmark	DBPC	11,200/daily/2 weeks; 5600/daily/10 weeks	12 weeks	placebo	8	8
Khan 2023 [37]	India	PIRT	60,000/weekly/2 months; 60,000/per month	3 months	no supplementation	47	45
Momeni 2016 [38]	Iran	DBPC	50,000/weekly	8 weeks	placebo	30	30
Muley 2019 [39]	India	RCT	60,000/weekly	8 weeks	n/a	40	30
Sadyia 2014 [40]	UAE	DBPC	6000/daily	3 months	placebo	45	42
Safarpour 2020 [41]	Iran	DBPC	50,000/weekly	8 weeks	placebo	42	43
Tabesh 2015 [42]	Iran	DBPC	50,000/weekly	8 weeks	placebo	29	30
Yiu 2013 [43]	Hong Kong	DBPC	5000/daily	12 weeks	placebo	50	50

**Table 2:** Specific parameters, their levels, and difference (%) vs. baseline after intervention of studies. Bold data represents parameters, which were positively affected by the treatment.

Name of the First Author and Year of Publication	Vitamin D Levels (Vitamin D Group) After Intervention [ng/mL]	Vitamin D Levels Diff vs. Baseline (%)	HbA1c Levels (Vitamin D Group) After Intervention (%)	HbA1c Levels Diff vs. Baseline (%)	SBP (Vitamin D Group) After Intervention [mmHg]	DBP (Vitamin D Group) After Intervention [mmHg]	SBP Diff vs. Baseline (%)	DBP Diff vs. Baseline (%)	PTH [pmol/L]	PTH Diff vs. Baseline (%)	Ca^2+^ [mmol/L]	Ca^2+^ Diff vs. Baseline (%)	Fasting Blood Glucose (FBG) [mmol/L]	FBG Diff vs. Baseline (%)	BMI (Vitamin D Group) Before Intervention	BMI (Vitamin D Group) After Intervention	BMI Levels Diff vs. Baseline (%)
Ahmadi 2013 [24]	71.23	**406.61 [↑]**	7.22	**1.26 [↑]**	119.67	n/a	**4.53 [↓]**	**n/a**	n/a	n/a	2.42	5.2[↑]	n/a	n/a	28.38	n/a	n/a
Al-Sofiani 2015 [25]	36.44	**229 [↑]**	7.85	**1.26 [↓]**	133	77	**7.96 [↓]**	**1.91 [↓]**	n/a	n/a	9.46	20.35 [↑]	8.9	5.31 [↓]	28.8	28.33	1.63 [↓]
Barale 2020 [26]	41.2	**372.48 [↑]**	6.36	**14.97 [↓]**	135	75	**14.56 [↓]**	**9.64 [↓]**	4.87	26.43 [↓]	2.4	4.34 [↑]	9.1	2.77 [↓]	29.7	28.7	3.37 [↓]
Behshad 2022 [27]	40.05	**120.78 [↑]**	7.94	**4.22 [↓]**	134.43	71.43	**0.22 [↓]**	**2.55 [↓]**	n/a	n/a	2.38	2.68 [↑]	8.84	4.17 [↓]	25.64	25.6	0.16 [↓]
Byrn 2019 [28]	53	**120.83 [↑]**	6.9	**4.16 [↓]**	n/a	n/a	**n/a**	**n/a**	n/a	n/a	2.42	1.04 [↑]	n/a	n/a	37.32	n/a	n/a
Cojic 2020 [29]	36.8	**73.65 [↑]**	6.68	**0.6 [↑]**	137.28	83.34	**0.31 [↑]**	**0.28 [↓]**	n/a	n/a	2.4	1.22 [↑]	7.34	7.44 [↓]	29.34	28.22	3.82 [↓]
Ebrahimkhani 2020 [30]	25.05	**149.25 [↑]**	n/a	**n/a**	120.6	74	**14.41 [↓]**	**18.41 [↓]**	n/a	n/a	n/a	n/a	n/a	n/a	30.2	29.9	0.99 [↓]
El Hajj 2020 [31]	34.9	**135.81 [↑]**	6.53	**0.91 [↓]**	140	86	**0.71 [↓]**	**1.91 [↑]**	3.25	18.13 [↓]	n/a	n/a	10.25	0.05 [↓]	22.6	21.2	6.19 [↓]
Elkassaby 2014 [32]	51.2	**117 [↑]**	6.1	**1.64 [↓]**	n/a	n/a	**n/a**	**n/a**	n/a	n/a	n/a	n/a	7	2.94 [↑]	30.6	n/a	n/a
Esfandiari 2019 [33]	37.63	**73.65 [↑]**	6.26	**1.57 [↓]**	n/a	n/a	**n/a**	**n/a**	n/a	n/a	2.14	3.25 [↑]	6.22	13.36 [↓]	n/a	n/a	n/a
Imanparast 2019 [34]	51.79	**194.6 [↑]**	8.38	**3.2 [↑]**	129.14	77.82	**1.53 [↓]**	**3.49 [↓]**	4.3	12.26 [↓]	2.39	3.78 [↓]	9.89	8.02 [↑]	28.29	28.55	0.92 [↑]
Jorde 2009 [35]	47.32	**97.16 [↑]**	7.8	**2.5 [↓]**	136.8	81	**0.94 [↑]**	**2.01 [↑]**	3.6	7.69 [↓]	2.32	1.27 [↓]	9.6	2.04 [↓]	32.8	32.6	0.61 [↓]
Kampmann 2014 [36]	41.96	**238.38 [↑]**	6.84	**0.58 [↓]**	134	74	**0.97 [↑]**	**1.33 [↓]**	5.9	4.83 [↓]	2.276	2.22 [↑]	7.81	1.42 [↑]	35.3	35.1	0.57 [↓]
Khan 2023 [37]	30.97	**121.05 [↑]**	7.21	**9.76 [↓]**	n/a	n/a	**n/a**	**n/a**	n/a	n/a	n/a	n/a	7.22	13.53 [↓]	23.75	23.85	0.42 [↑]
Momeni 2016 [38]	35.78	**143.3 [↑]**	8.02	**2.55 [↓]**	n/a	n/a	**n/a**	**n/a**	n/a	n/a	n/a	n/a	8.48	4.11 [↑]	n/a	n/a	n/a
Muley 2019 [39]	43.6	**260.33 [↑]**	8.3	**3.48 [↓]**	134.8	81.5	**4.8 [↓]**	**4.11 [↓]**	n/a	n/a	n/a	n/a	n/a	n/a	27.9	27.8	0.36 [↓]
Sadyia 2014 [40]	30.88	**170.87 [↑]**	8.2	**0**	133	74	**3.9 [↑]**	**5.71 [↑]**	5	15.25 [↓]	2.31	0.00	9.8	5.37 [↑]	37.9	n/a	n/a
Safarpour 2020 [41]	38.86	**125.4 [↑]**	6.76	**9.98 [↓]**	n/a	n/a	**n/a**	**n/a**	n/a	n/a	n/a	n/a	9.72	1.76 [↑]	30.43	30.34	0.3 [↓]
Tabesh 2015 [42]	34.92	**214.02 [↑]**	n/a	**n/a**	115.6	78	**5.24 [↓]**	**3.7 [↓]**	n/a	n/a	n/a	n/a	n/a	n/a	30.5	30.48	0.07 [↓]
Yiu 2013 [43]	58.6	**177.72 [↑]**	7.3	**0.68 [↓]**	145	81	**0.69 [↑]**	**1.25 [↑]**	4.1	12.95 [↓]	n/a	n/a	7.37	2.64 [↑]	25.8	25.9	0.04[↑]

Abbreviations: glycated hemoglobin (HbA1C), systolic (SBP), diastolic (DBP), parathormone (PTH), serum calcium (Ca^2+^), fasting blood glucose (FBG), body mass index (BMI). [↓] and [↑] represent whether the level was decreased or increased vs. baseline.

**Table 3:** Conclusions of included studies.

Name of the First Author and Year of Publication	Conclusion
Ahmadi 2013 [24]	Vitamin D supplementation for three months did not reduce proteinuria in diabetic patients [24].
Al-Sofiani 2015 [25]	Vitamin D supplementation in T2DM patients with low vitamin D improved their vitamin D levels and reduced proteinuria but did not affect blood sugar control [25].
Barale 2020 [26]	Vitamin D supplementation improved blood sugar, HbA1c, blood pressure, and cholesterol in poorly controlled T2DM patients with vitamin D deficiency [26].
Behshad 2022 [27]	High-dose vitamin D supplementation raised vitamin D levels and slightly improved blood sugar control in the study [27].
Byrn 2019 [28]	High and low-dose vitamin D supplementation had no significant difference in cognitive function [28].
Cojic 2020 [29]	Vitamin D supplementation may improve blood vessel function in T2DM patients on metformin by reducing harmful inflammation and oxidative stress [29].
Ebrahimkhani 2020 [30]	Vitamin D supplementation and curcuminoids helped improve body composition and blood pressure in people with T2DM and vitamin D deficiency [30].
El Hajj 2020 [31]	Vitamin D supplementation led to a decrease in some inflammatory markers in T2DM [31].
Elkassaby 2014 [32]	High-dose vitamin D supplementation improves glycemia transiently, but the biological significance is questionable [32].
Esfandiari 2019 [33]	Vitamin D supplementation can be regarded as an effective way to prevent the progression of diabetic nephropathy by reducing levels of inflammatory markers—IL-6 and TNF-a and proteinuria [33].
Imanparast 2019 [34]	Vitamin D supplementation and chromium are presumably valuable for controlling and preventing the complications of diabetes by reducing the Homeostatic Model Assessment for Insulin Resistance, which decreases TNF-α [34].
Jorde 2009 [35]	Vitamin D supplementation did not significantly affect glucose metabolism in subjects with T2DM but without vitamin D deficiency [35].
Kampmann 2014 [36]	Vitamin D supplementation might increase insulin secretion in patients with established T2DM but does not improve insulin resistance, blood pressure, inflammation, or HbA1c [36].
Khan 2023 [37]	Vitamin D supplementation can improve the glycemic parameters of T2DM with concurrent vitamin D deficiency [37].
Momeni 2016 [38]	Vitamin D supplementation in T2DM patients with vitamin D deficiency or insufficiency leads to normalization of serum vitamin D levels and a decrease in proteinuria compared to the control group, but it did not improve glycemic control indices in the patients [38].
Muley 2019 [39]	Vitamin D supplementation improved the anthropometric and lipid parameters among the subjects, thus suggesting a beneficial role in the cardio-metabolic profile of the T2DM subjects [39].
Sadyia 2014 [40]	Vitamin D supplementation for obese T2DM patients who are vitamin D deficient normalized the vitamin D status. It reduced the incidence of eucalcemic parathyroid hormone elevation but showed no effect on metabolic control [40].
Safarpour 2020 [41]	Vitamin D supplementation may improve T2D by decreasing HbA1c and increasing SIRT1 and irisin in vitamin D-deficient T2D patients [41].
Tabesh 2015 [42]	Vitamin D supplementation, in combination with calcium, demonstrated beneficial effects on BMI, hip circumference, and systolic blood pressure in patients with T2DM and vitamin D insufficiency [42].
Yiu 2013 [43]	Vitamin D supplementation did not significantly affect vascular function or serum biomarkers of inflammation and oxidative stress in patients with type 2 DM after 12 weeks of oral supplementation [43].

## Data Availability

The original data presented in the study are openly available in SCOPUS, Web of Science and PubMed databases at www.scopus.com, www.webofscience.com and www.pubmed.ncbi.nlm.nih.gov.

## Abbreviations

T2DM	type 2 diabetes mellitus
25(OH)D	calcifediol, as calcidiol or 25-hydroxycholecalciferol
FBG	fasting blood glucose
HbA1c	glycated hemoglobin
PTH	parathormone
VDR	vitamin D receptor
RCT	randomized controlled trial
e.g.,	for example
VD	vitamin D
Ca^2+^	calcium
BP	blood pressure
DBPC	double-blind, placebo-controlled
RC	randomized controlled
DBACC	double-blind, active comparator-controlled
RPCT	randomized placebo-controlled trial
PIRT	prospective interventional randomized trial
IU	international unit
PRISMA	preferred reporting items for systematic reviews and Meta-Analyses
BMI	body mass index
SBP	systolic blood pressure
DBP	diastolic blood pressure
UAE	United Arab Emirates
D3	cholecalciferol
PTH	parathormone
CAMP	cathelicidin antimicrobial peptide
Th cells	T-helper cells
Treg cells	T-regulatory cells
IgG	immunoglobulin G
IgM	immunoglobulin M
IFN γ	interferon gamma
TNF-α	tumor necrosis factor-alpha
IL-2	interleukin 2
IL-4	interleukin 4
IL-5	interleukin 5
IL-6	interleukin 6
IL-10	interleukin 10
IL-13	interleukin 13
IL-17	interleukin 17
IL-21	interleukin 21
